# Best practices: Appropriate use of the new β-lactam/β-lactamase inhibitor combinations, ceftazidime-avibactam and ceftolozane-tazobactam in South Africa

**DOI:** 10.4102/sajid.v37i1.453

**Published:** 2022-10-20

**Authors:** Adrian J. Brink, Jennifer Coetzee, Guy A. Richards, Charles Feldman, Warren Lowman, Hafsah D. Tootla, Malcolm G.A. Miller, Abraham J. Niehaus, Sean Wasserman, Olga Perovic, Chetna N. Govind, Natalie Schellack, Marc Mendelson

**Affiliations:** 1 Division of Medical Microbiology, Institute of Infectious Diseases and Molecular Medicine, Faculty of Health Sciences, University of Cape Town, Cape Town, South Africa; 2 Division of Microbiology, Ampath National Reference Laboratory, Centurion, South Africa; 3 Faculty of Health Sciences, University of the Witwatersrand, Johannesburg, South Africa; 4 Department of Internal Medicine, Faculty of Health Sciences, University of the Witwatersrand, Johannesburg, South Africa; 5 Department of Clinical Microbiology, Pathcare/Vermaak Pathologists, Gauteng, South Africa, South Africa; 6 Department Clinical Microbiology and Infectious Diseases, Faculty of Health Sciences, University of Witwatersrand, Johannesburg, South Africa; 7 Department of Clinical Microbiology and Infection Prevention and Control, Wits Donald Gordon Medical Centre, Johannesburg, South Africa; 8 Division of Medical Microbiology, National Health Laboratory Service, Red Cross War Memorial Children’s Hospital, Cape Town, South Africa; 9 Division of Critical Care, Department of Anaesthesia and Perioperative Medicine, Faculty of Health Sciences, University of Cape Town, Cape Town, South Africa; 10 Department of Medical Microbiology, Ampath Laboratory Services, Cape Town, South Africa; 11 Division of Infectious Diseases and HIV Medicine, Department of Medicine, Groote Schuur Hospital, University of Cape Town, Cape Town, South Africa; 12 Wellcome Centre for Infectious Diseases Research in Africa, Institute of Infectious Disease and Molecular Medicine, University of Cape Town, Cape Town, South Africa; 13 National Institute for Communicable Disease, National Health Laboratory Services, Johannesburg, South Africa; 14 School of Pathology, Clinical Microbiology and Infectious Diseases, Faculty of Health Sciences, University of the Witwatersrand, Johannesburg, South Africa; 15 Department of Medical Microbiology, Lancet Laboratories, KwaZulu-Natal, Durban, South Africa; 16 Antimicrobial Research Unit, School of Health Sciences, University of KwaZulu-Natal, Durban, South Africa; 17 Department of Pharmacology, Faculty of Health Sciences, University of Pretoria, Pretoria, South Africa

**Keywords:** antimicrobial stewardship, β-lactam/β-lactamase inhibitor combinations, ceftazidime-avibactam, ceftolozane-tazobactam, Gram-negatives, *Pseudomonas aeruginosa*, Enterobacterales

## Abstract

Antibiotic stewardship of hospital-acquired infections because of difficult-to-treat resistant (DTR) Gram-negative bacteria is a global challenge. Their increasing prevalence in South Africa has required a shift in prescribing in recent years towards colistin, an antibiotic of last resort. High toxicity levels and developing resistance to colistin are narrowing treatment options further. Recently, two new β-lactam/β-lactamase inhibitor combinations, ceftazidime-avibactam and ceftolozane-tazobactam were registered in South Africa, bringing hope of new options for management of these life-threatening infections. However, with increased use in the private sector, increasing levels of resistance to ceftazidime-avibactam are already being witnessed, putting their long-term viability as treatment options of last resort, in jeopardy. This review focuses on how these two vital new antibiotics should be stewarded within a framework that recognises the resistance mechanisms currently predominant in South Africa’s multi-drug and DTR Gram-negative bacteria. Moreover, the withholding of their use for resistant infections that can be treated with currently available antibiotics is a critical part of stewardship, if these antibiotics are to be conserved in the long term.

## Introduction

The prevalence of infections caused by difficult-to-treat resistant Gram-negative bacteria (DTR-GNB) is rapidly increasing, and along with constantly evolving epidemiology, represents a major challenge to the management of hospital-acquired infections (HAIs).^1^ In this regard, DTR describes treatment-limiting resistance to all first-line agents, that is, all β-lactams, including carbapenems and previous generation β-lactamase inhibitor combinations (BLICs), and fluoroquinolones.^2^ Arguably, the greatest threat from DTR-GNB comes in the form of carbapenem-resistant Enterobacterales (CRE) such as carbapenemase-producing *Klebsiella pneumoniae*.^3^

There has been an unprecedented proliferation of carbapenemase encoding genes, the evolutionary epidemiology and resistome dynamics of which were recently highlighted in several local studies.^4,5,6^ Widespread transmission is reported, and is mediated by clonal, multiclonal and horizontal mechanisms.^4,5,6^ Enhanced surveillance for CRE causing blood-stream infections (BSIs) in South Africa, recently demonstrated a significant increase in the proportion of cases especially from Gauteng and the Western Cape.^4^

Moreover, the proportion of CRE isolates, specifically *K. pneumoniae*, phenotypically non-susceptible to class 2 carbapenems (imipenem, meropenem and doripenem) ranges from 50% to 53%, while non-susceptibility to ertapenem is 86%, reflecting production of diverse carbapenemases, with a predominance of OXA-48, and reduced permeability (porin reduction or alteration). The dire situation with regard to treatment options is reflected by the fact that up to 22% and 13% of CRE isolates are also non-susceptible to tigecycline and colistin, respectively, and the overall in-hospital mortality regardless of therapy is 38%.^4^

Available options for the treatment of HAIs caused by CR-*Pseudomonas aeruginosa* (*P. aeruginosa*) are also scarce, and recent reports emphasising the spread of colistin non-susceptibility in environments with high volumes of colistin or polymyxin usage, are a major concern.^3^ South Africa is no exception, with CR among *P. aeruginosa* isolated from bacteraemic patients in the public and private sectors, ranging from 30% to 40%, while concurrent colistin resistance has emerged and susceptibility to this antibiotic of last resort has decreased year on year.^7,8^ Similarly, agents to which DTR-*Acinetobacter baumannii* are susceptible are very few in number and there is a paucity of new antibiotics in the pipeline for these pathogens.

A major challenge is how best to optimise care for infections caused by DTR-GNB in the context of diminishing antibiotic options that may be less effective and associated with higher toxicity. There is a need for appropriate and effective initial therapy while promoting antibiotic stewardship (ABS) principles, emphasising the continued use of existing antibiotics to which the bacteria remain susceptible.^1^ In this regard, it is evident that new molecules and novel BLICs with *in vitro* activity against DTR-GNB, only partially address the currently prevalent mechanisms of resistance (Table 1).^9,10^ As such, effective therapy is dependent on accurate and prompt identification of the organism, and on phenotypic and genotypic antimicrobial susceptibility and mechanisms of resistance testing, respectively.

**TABLE 1:** List of the new agents for difficult-to-treat resistant Gram-negatives.†

Antibiotic	ESBL	KPC	OXA-48	MBL	CRPA	CRAB
**β-lactam/β-lactamase inhibitors**						
Meropenem/vaborbactam	✓	✓	-	-	-	-
Meropenem/nacubactam	✓	✓	✓	-	-	-
Meropenem/QPX7728	✓	✓	✓	✓	±	✓
Imipenem/relebactam	✓	✓	-	-	✓	-
Ceftazidime/avibactam†	✓	✓	✓	-	✓	-
Ceftolozane/tazobactam†	✓	-	-	-	✓	-
Cefepime/tazobactam	✓	-	✓	-	-	-
Cefepime/enmetazobactam	✓	-	-	-	-	-
Cefepime/zidebactam	✓	✓	✓	✓	±	±
Cefepime/VNRX5133	✓	✓	✓	±	±	-
Cefepime/QPX7728	✓	✓	✓	✓	±	-
Ceftibuten/VNRX-7145	✓	-	-	-	-	-
Ceftibuten/QPX7728	✓	✓	✓	-	-	-
Cefpodoxime/ETX-0282	✓	-	-	-	-	-
Aztreonam/avibactam	✓	✓	✓	✓	-	-
Sulbactam/durlobactam	✓	✓	✓	✓	-	✓
**β-lactams**						
Cefiderocol	✓	✓	✓	±	✓	±
Tebipenem	✓	-	-	-	-	-
Sulopenem	✓	-	-	-	-	-
**Aminoglycosides**						
Plazomicin	✓	✓	✓	±	-	-
**Tetracyclines**						
Eravacycline	✓	✓	✓	✓	-	±
**Polymyxins**						
SPR741 plus β-lactams	✓	±‡	±‡	-	-	✓
SPR206	✓	✓	✓	✓	✓	✓
QPX9003	✓	✓	✓	✓	✓	✓

*Source*: Paterson DL, Isler B, Stewart A. New treatment options for multiresistant gram negatives. Curr Opin Infect Dis. 2020;33(2):214–223. https://doi.org/10.1097/QCO.0000000000000627

ESBL, extended spectrum β-lactamases; KPC, *Klebsiella pneumoniae* carbapenemase; OXA-48, Oxacillinase-48; MBL, Metallo-beta-lactamases; CRPA, Carbapenem-resistant *Pseudomonas aeruginosa*; CRAB, Carbapenem-resistant *Acinetobacter baumannii.*

† , Only ceftazidime-avibactam and ceftolozane-tazobactam are registered in South Africa.

‡ , Active against *Escherichia coli*, inactive against *Klebsiella pneumoniae*.

Ceftazidime-avibactam (CA) and ceftolozane-tazobactam (CT) are the first of the new generation BLICs registered in South Africa. The potential for uncontrolled use of these agents as a ‘one-size fits all’ treatment for DTR-GNB will prove catastrophic for their future preservation as viable therapeutic options. Ceftolozane-tazobactam and CA were previously prescribed in South Africa as section 21 drugs since 2018, and utilisation data indicates a sharp increase in the compound annual growth rate, specifically a 383% increase of CA consumption, over the past three years (Data on file). The utilisation is almost exclusively driven by the private sector, where CA is also the dominant agent of the two, with an overall 100% market share. The global temporary recall of CT at the beginning of 2021 by the manufacturer, as well as other stock issues relating to this new BLIC are some of the reasons for exclusive CA consumption.

The objectives of the article’s recommended approach to the management of MDR- and DTR-GNB (i.e. mechanism-based inhibition therapy) are to:

- Optimise patient outcomes in settings where there has been increasing dependence on colistin as salvage monotherapy.
- Avoid redundant and inappropriate use of CA and CT, from an ABS and cost-containment point-of-view.
- Ensure the longevity of existing broad-spectrum agents (i.e. carbapenems, ceftazidime, cefepime, tigecycline, piperacillin-tazobactam and colistin) and the new antibiotics (i.e. CT and CA and those that follow).

## Mechanisms of resistance as a confounder in antibiotic stewardship

The mechanisms by which GNB develop and express resistance, may interfere with several facets of ABS and impact on clinical treatment pathways, including the choice of an empiric antibiotic regimen, the potential for de-escalation, and the management of clinical failure because of the emergence of resistance on therapy.^11,12^ Antibiotic resistance in GNB results from the expression of antibiotic-inactivating enzymes and non-enzymatic mechanisms, both of which may be intrinsically expressed by a given species (chromosomal genes) or acquired by a subset of strains as a consequence of two distinct, albeit not mutually exclusive, genetic events^3^:

- Mutations in chromosomal genes resulting in an increase in the expression of intrinsic resistance mechanisms, such as antibiotic-inactivating enzymes, efflux pumps, permeability alterations mediated by loss of outer membrane porins or target modifications. A key feature of these determinants is that exposure to a given class may also select mutants with resistance to other often unrelated antibiotic classes.
- Horizontal transfer of mobile genetic elements carrying resistance genes, most notably plasmids encoding for β-lactamases (such as carbapenemases), and aminoglycoside-modifying enzymes, or non-enzymatic mechanisms resulting in fluoroquinolone resistance.

In non-fermenting GNB such as *P. aeruginosa*, MDR and DTR may emerge following sequential chromosomal mutations, which may lead to the overproduction of intrinsic β-lactamases such as AmpC and hyperexpression of efflux pumps, target modifications and permeability alterations.^3,7^ *P. aeruginosa* also has the ability to acquire mobile genetic elements encoding for resistance determinants, including carbapenemases such as VIM, which is particularly prevalent in SA.^7,13,14,15^ The spontaneous mutation rate for expression of resistance may occur as frequently as 1 in 10^6^ to 1 in 10^7^ wild type strains. This process may be accelerated by the use of antibiotics with anti-pseudomonal activity, particularly if therapy is prolonged.^16^

With this in mind, ABS should preferably involve the application of certain fundamental tenets when selecting antibiotics for treatment of DTR-GNB, chief among which are the following:

- Early source control is critical to a good outcome. Depending on the source, this may involve debridement, laparotomy, thoracotomy, medical device removal (e.g. urine catheters, intravenous/intra-arterial catheters) and drainage of abscesses.
- Antibiotics with anti-pseudomonal activity (e.g. ceftazidime, cefepime, piperacillin-tazobactam, imipenem, meropenem and ciprofloxacin) should preferably be restricted to those patients at risk for (empiric therapy), or infected with (directed therapy) *P. aeruginosa.*
- If risk factors for pseudomonal infection are absent, agents with minimal or no anti-pseudomonal activity (e.g. amoxicillin-clavulanate, ceftriaxone and ertapenem) would be preferred.^16^

## Conceptual approach to mechanism-based therapy

The available evidence regarding the use of currently available BLICs, as well as an emerging body of data on novel non-β-lactam agents in the antibiotic development pipeline, require a paradigm shift with regard to how, we recommend the use of both currently available and new antibiotics.

Translating microbiological data relating to β-lactamase genealogy in South Africa into clinical practice, and enablement of this information at the bedside remain a formidable challenge. β-lactamases such as OXA-48 (and its variants) and NDM-1, either alone, or simultaneously present on plasmids, are the predominant carbapenemases among Enterobacterales with very few, or no *K. pneumoniae* carbapenemases (KPC), or other carbapenemases evident.^4,5,6^ However, recent unpublished data suggest a steady increase in KPC in Gauteng province. In *P. aeruginosa-*BSIs in South Africa,^7^ metallo-β-lactamases (MBL) such as VIM and the Ambler class C AmpC enzymes predominate, and in addition, MDR efflux pumps are expressed in the majority of the non-susceptible isolates investigated.

**FIGURE 1:**
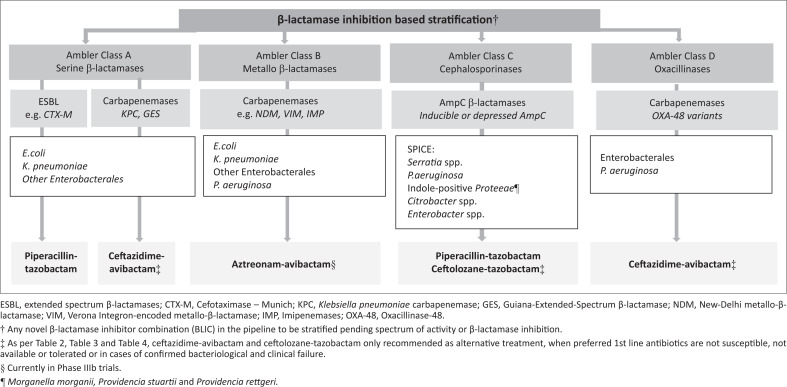
Conceptual approach to β-lactamase inhibitor therapy for severe infections.

Therefore, recommendations for antibiotic use should be strategic and should rely, in the case of new BLICs, on categorisation by ability to inhibit specific β-lactamases, when directed therapy is required (Figure 1). Hence, in order to minimise antibiotic selective pressure, a ‘Best Practice’ guide for existing and new antibiotic options in the South African context (Table 2, Table 3 and Table 4), which includes dose and administration strategies for critically ill patients, is proposed (Table 5).^17,18^

**TABLE 2:** Recommended intravenous antibiotic treatment options for extended-spectrum β-lactamase producing Enterobacterales.†,‡,§

Source	Preferred treatment	Alternative treatment§§
**Pyelonephritis or complicated UTI¶**		
	Aminoglycosides or Fluoroquinolones (ciprofloxacin, levofloxacin) or Piperacillin-tazobactam†† or Ertapenem	Meropenem or Imipenem-cilastatin
**Infections outside of the urinary tract**		
Complicated intra-abdominal infections	Tigecycline or Piperacillin-tazobactam†† or Ertapenem	Meropenem or Imipenem-cilastatin
Pneumonia	Piperacillin-tazobactam†† or Ertapenem	Meropenem or Imipenem-cilastatin
Bloodstream infections	Aminoglycosides‡‡ or Ertapenem	Meropenem or Imipenem-cilastatin

UTI, urinary tract infections; BLIC, β-lactamase inhibitor combinations; MIC, minimum inhibitory concentration.

† , Assuming *in vitro* susceptibility to agents in the table.

‡ , Refer to Table 5 for recommended dosing and administration schedules for adult critically ill patients with normal renal function.

§ , From a strategic point-of-view, ceftazidime-avibactam and ceftolozane-tazobactam are not recommended for extended-spectrum β-lactamase producing Enterobacterales infections.

¶ , Complicated UTI, is defined as ‘UTI that occurs in association with a structural or functional abnormality of the genitourinary tract, or any UTI in a male patient’. This excludes UTIs in catheterised patients or caused by a resistant bacterium.

†† , Only if MIC ≤ 4.

‡‡ , If extended-spectrum β-lactamase producing Enterobacterales bloodstream infection is secondary to urinary source.

§§ , If 1st line options are not susceptible, not available or tolerated or in cases of confirmed bacteriological and clinical failure.

**TABLE 3:** Recommended intravenous antibiotic treatment options for AmpC producing bacteria.†,‡

Source	Preferred treatment	Alternative treatment¶¶
***Pseudomonas aeruginosa***		
Pyelonephritis or complicated UTI§	Ciprofloxacin or Aminoglycosides or Ceftazidime or Cefepime or Piperacillin-tazobactam	Meropenem or Imipenem-cilastin or Colistin or polymyxin B
Infections outside of the urinary tract	Ceftazidime or Cefepime or Piperacillin-tazobactam or Meropenem or Imipenem-cilastatin or Colistin or polymyxin B	Aminoglycosides (Limited to uncomplicated bloodstream infections with complete source control¶) or Ceftolozane-tazobactam††
**Enterobacterales (SPICE)‡‡**		
Pyelonephritis or complicated UTI§	Ciprofloxacin or Aminoglycosides or Piperacillin-tazobactam or Cefepime or Ertapenem	Meropenem or Imipenem-cilastin
Biliary sepsis	Piperacillin-tazobactam or Cefepime or Ertapenem	Meropenem or Imipenem-cilastin or Ceftolozane-tazobactam††
Complicated intra-abdominal infections	Tigecycline§§ or Piperacillin-tazobactam or Cefepime or Ertapenem	Meropenem or Imipenem-cilastin or Ceftolozane-tazobactam††
Pneumonia	Piperacillin-tazobactam or Cefepime or Ertapenem	Meropenem or Imipenem-cilastin or Ceftolozane-tazobactam††
Primary bacteraemia	Ertapenem	Meropenem or Imipenem-cilastin or Ceftolozane-tazobactam††

UTI, urinary tract infection; SPICE, *Serratia* spp., *P. aeruginosa*, indole-positive Proteeae (*Morganella morganii, Providencia rettgeri* and *Providentia stuartii*), *Citrobacter* spp., and *Enterobacter* spp.; XDR, extensive drug-resistant; PDR, pan-drug-resistant.

† , Assuming *in vitro* susceptibility to agents in the table.

‡ , Refer to Table 5 for recommended dosing and administration schedules for adult critically ill patients with normal renal function.

§ , Complicated UTI, is defined as ‘UTI that occurs in association with a structural or functional abnormality of the genitourinary tract, or any UTI in a male patient’. This excludes UTIs in catheterised patients or caused by a resistant bacterium.

¶ , Uncomplicated bloodstream infections include a bloodstream infection that is because of a urinary source or a catheter-related bloodstream infection with removal of the infected vascular catheter.

†† , Ceftolozane-tazobactam only in difficult-to-treat resistant infections that is, as an antibiotic of last resort.

‡‡ , Very little data to guide colistin or polymyxin combination therapy or otherwise for AmpC-producing Enterobacterales is available and therefore these antibiotics have not been included.

§§ , *Morganella* spp., *Proteus* spp. and *Providencia* spp. are inherently resistant to tigecycline.

¶¶ , If 1st line options are not susceptible, not available or tolerated or in cases of confirmed bacteriological and clinical failure.

**TABLE 4:** Recommended intravenous antibiotic treatment options for carbapenem-resistant Enterobacterales.†,‡

Source	Preferred treatment	Alternative treatment¶¶¶
**Pyelonephritis or complicated UTI§**		
	Aminoglycosides or Ciprofloxacin	Meropenem or imipenem-cilastatin
**Infections outside of the urinary tract¶**		
Biliary sepsis	Meropenem or imipenem-cilastatin†† plus 2nd active antibiotic or Tigecycline§§ with/out 2nd active antibiotic or Colistin or polymyxin B¶¶ plus 2nd active antibiotic	Ceftazidime-avibactam (OXA-48, KPC, GES)‡‡ or Aztreonam plus Ceftazidime-avibactam (NDM, IMP, VIM)‡‡
Complicated intra-abdominal infections	Meropenem or imipenem-cilastatin†† plus 2nd active agent or Tigecycline§§ plus 2nd active antibiotic or Colistin or polymyxin¶¶ plus 2nd active antibiotic	Ceftazidime-avibactam (OXA-48, KPC, GES)‡‡,††† or Aztreonam plus Ceftazidime-avibactam (NDM, IMP, VIM)‡‡,†††
Pneumonia	Meropenem or imipenem-cilastatin†† plus 2nd active agent or Colistin or polymyxin B¶¶ plus 2nd active antibiotic	Ceftazidime-avibactam (OXA-48, KPC, GES)‡‡ or Aztreonam plus Ceftazidime-avibactam (NDM, IMP, VIM)‡‡
Primary bacteraemia	Meropenem or imipenem-cilastatin†† plus 2nd active agent or Colistin or polymyxin B¶¶ plus 2nd active antibiotic	Ceftazidime-avibactam (OXA-48, KPC, GES)‡‡ or Aztreonam plus Ceftazidime-avibactam (NDM, IMP, VIM)‡‡

UTI, urinary tract infections; OXA-48, Oxacillinase-48; MBL, Metallo-β-lactamase; NDM, New-Delhi metallo-β-lactamase; VIM, Verona Integron-encoded metallo-β-lactamase; IMP, Imipenemases; KPC, *Klebsiella pneumoniae* carbapenemase; MIC, minimum inhibitory concentration.

† , Assuming *in vitro* susceptibility to agents in the table.

‡ , Refer to Table 5 for recommended dosing and administration schedules for adult critically ill patients with normal renal function.

§ , Complicated UTI, is defined as ‘UTI that occurs in association with a structural or functional abnormality of the genitourinary tract, or any UTI in a male patient’. This excludes UTIs in catheterised patients or caused by a resistant bacterium.

¶ , In certain clinical scenarios, and requiring close monitoring for clinical response, monotherapy with an active agent may be considered by a stewardship team.

†† , According to carbapenem MIC and reported as susceptible. Generally, if MIC reported as ≤8 mg/L combine with another active agent while if MIC ≤ 2 mg/L, monotherapy at optimised dosing, may be considered in selected cases.

‡‡ , Ceftazidime-avibactam only in difficult-to-treat infections that is, as an antibiotic of last resort.

§§ , *Morganella* spp., *Proteus* spp. and *Providencia* spp. are inherently resistant to tigecycline.

¶¶ , *Proteus* spp., *Serratia marcescens, Providencia* spp. and *Morganella morganii* are inherently resistant to colistin and polymyxin B.

††† , Complicated intra-abdominal infections are the indication with the most scope for inappropriate use of ceftazidime-avibactam and resistance emerging relating to prolonged use without adequate source control.

¶¶¶ , If 1st line options are not susceptible, not available or tolerated or in cases of confirmed bacteriological and clinical failure.

**TABLE 5:** Recommended intravenous antibiotic dosing and administration schedules for critically ill adult patients (with normal renal function).†

Agent	Dosing and administration schedules
**Aminoglycosides** ‡	
Amikacin	15 mg/kg – 30 mg/kg once daily
Gentamicin	7 mg/kg once daily
Tobramycin	7 mg/kg once daily
Β**LICs**	
Ceftazidime-avibactam	2.5 g 8-hourly, infused over 2 h
Ceftolozane-tazobactam	3 g 8-hourly, infused over 1 h
Piperacillin-tazobactam	4.5 g loading§, 4.5 g 6-hourly infused over 3 h or 18 g infused over 24 h
**Carbapenems**	
Ertapenem	1 g once-daily or 12-hourly, infused over 1 h
Doripenem¶	1 g 8-hourly, infused over 4 h
Imipenem	1 g 6–8 hourly, infused over 1–3 h
Meropenem	2 g 8–hourly, infused over 3 h
**Cephalosporins**	
Cefepime	2 g loading§, 2 g 8-hourly infused over 4 h or 6 g infused over 24 h
Ceftazidime	2 g loading§, 2 g 8-hourly infused over 4 h or 6 g infused over 24 h
**Fluoroquinolones**	
Ciprofloxacin	400 mg 8-hourly
Levofloxacin	750 mg once daily
**Polymyxins**	
Colistin††	9 MU – 12 MU loading, 3 MU 8-hourly or 4.5 MU 12-hourly (60 kg)
Polymyxin B‡‡	20 000 IU/kg – 25 000 IU/kg (2 mg/kg – 2.5 mg/kg) loading dose and 12 500 IU/kg – 15 000 IU/kg (1.25 mg/kg – 1.5 mg/kg) 12-hourly
**Tigecycline**	200 mg loading§, 100 mg 12-hourly

BLICs: β-lactam β-lactamase inhibitor combinations.

† , Maximum dosing to account for pharmacokinetic disturbances in this population and to target the upper end of clinical breakpoints.

‡ , Subsequent doses and dosing interval should be based on pharmacokinetic evaluation and use of therapeutic drug monitoring.

§ Typically, maintenance doses are begun 30 min – 1 h following administration of the loading dose.

¶ , Doripenem at standard doses has no advantage over group 2 carbapenems for susceptible Gram-negatives.

†† , For renal impairment, continuous renal replacement therapy and haemodialysis refer to the consensus colistin guideline, South Africa.^18^

‡‡ , Polymyxin B, unlike colistin, is administered in its active state and not as a prodrug. Hence it has superior pharmacological properties with less pharmacokinetic variability and dosing that is independent of renal function.

## Extended-spectrum β-lactamase-producing Enterobacterales

Some β-lactamase inhibitors inhibit ESBLs. Although hyperproduction of β-lactamases or additional resistance mechanisms may hamper the activity of these compounds, the previous generation BLICs such as piperacillin-tazobactam, remain active *in vitro* against a considerable proportion of ESBL-producing GNB. However, the use of non-carbapenem β-lactams for the treatment of ESBL infections has yielded conflicting and controversial results.^19,20,21^

Until the results of the MERINO trial (piperacillin-tazobactam versus meropenem for the treatment of BSIs caused by ceftriaxone-resistant *Escherichia coli* or *K. pneumoniae*) were published,^20,21^ based on previous comparative studies and reviews that reported the potential role of BLICs in treatment of serious ESBL-infections, piperacillin-tazobactam appeared to be a reasonable option for:^22,23^

- Low- to moderate-severity infections in non-critically ill patients.
- Infections resulting from urinary or biliary sources.
- Infections with piperacillin minimum inhibitory concentration (MICs) ≤ 4 µg/mL.

In the MERINO trial, however, 30-day mortality was 12.3% in patients randomised to piperacillin-tazobactam versus 3.7% in those randomised to meropenem.^20,21^ Nevertheless, the results of the MERINO study have been shown to have numerous biases. Henderson et al.^24^ reviewed the association of piperacillin-tazobactam and meropenem MICs and β-lactam resistance genes with mortality in the MERINO trial. According to reference broth microdilution, almost 20% of isolates were incorrectly categorised as susceptible, highlighting issues with piperacillin-tazobactam susceptibility testing itself. A high combined prevalence of OXA-1 β-lactamase and ESBLs was also demonstrated. Differences in outcome may also have related to the varying piperacillin-tazobactam dosing and administration schedules used.^22,25^

Therefore, piperacillin-tazobactam may be a carbapenem-sparing agent for BSIs as directed therapy, if the criteria are met, and dose is optimised and response to treatment is closely monitored (Table 5). Regarding the new BLICs, given its limited utility in South Africa because of the high prevalence of metallo-β lactamases, specifically VIM in *P. aeruginosa*, CT should be investigated as a carbapenem-sparing strategy for serious ESBL infections.

Regarding carbapenems, ertapenem as a class 1 carbapenem with no, or limited, activity against non-fermentative GNB including *Pseudomonas*, remains particularly suitable for ESBL-GNB infections.^26^ This includes BSI, as recently highlighted in a multinational retrospective cohort study (INCREMENT) in which comparable outcomes, for empiric and targeted therapy of mono-microbial BSI because of ESBL-producing pathogens, were seen with ertapenem and the broader spectrum carbapenems.^27^ The advantage of recommending ertapenem as a treatment option in this setting is that the drug does not appear to increase antibiotic resistance in *P. aeruginosa*.^28,29,30^

For patients with BSI because of ESBL-producing Enterobacterales, with severe infection, imipenem or meropenem instead of ertapenem has been recommended.^31^ However, this remains contentious with recent studies not demonstrating differences in outcomes, even in patients with septic shock.^32^ The distinct pharmacokinetic differences between ertapenem and the other carbapenems may account for conflicting findings and, therefore, choice of carbapenem in critically ill patients should best be decided by multi-disciplinary teams or institutional guidelines.

## AmpC-producing Gram-negative bacteria

AmpC resistance can be classified into three categories^33^:

- Inducible chromosomal resistance that emerges *in vivo* when in the presence of a β-lactam compound [historically referred to as ‘SPICE’ genera [*Serratia* spp., *P. aeruginosa,* indole-positive Proteeae (*Morganella morganii, Providencia rettgeri* and *Providentia stuartii*), *Citrobacter* spp. and *Enterobacter* spp.]
- Stable de-repression because of mutations in the AmpC regulatory genes (e.g. certain *E. coli* and *Shigella* spp.)
- The presence of plasmid-mediated AmpC genes that produce these β-lactamases regardless of the presence of a β-lactam antibiotic (e.g. certain *E. coli, K. pneumoniae* and *Salmonella* spp.).

It is important to note, that infections that pose a management conundrum, relate to the emergence of clinically relevant AmpC expression during antibiotic treatment, which has most frequently been described for *Enterobacter cloacae, Klebsiella aerogenes* (formerly *Enterobacter aerogenes*) and *Citrobacter freundii*.^33,34^ In this regard, few studies have provided reliable insights into effective management approaches for infection caused by such AmpC-producers. Nonetheless, it may be prudent to avoid third generation cephalosporins for the treatment of these organisms posing the greatest risk of AmpC induction. This has best been described in the context of especially, *E. cloacae* infections, even if susceptible *in vitro*.

In contrast, piperacillin-tazobactam and cefepime are potential directed therapeutic options in the treatment of susceptible AmpC producing bacteria. Both piperacillin and tazobactam are weak inducers of Amp-C β-lactamases.^33,34^ Cefepime has the advantage of being a weak inducer while withstanding hydrolysis by AmpC β-lactamases because of the formation of a stable acyl enzyme complex. As such the available clinical evidence does not support the use of alternative agents over piperacillin-tazobactam or cefepime, nor that resistance should be inferred (reporting an agent as resistant on the antibiogram) based on the Amp-C phenotype if piperacillin-tazobactam or cefepime is susceptible *in vitro*.^34,35^ Based on a systematic review and meta-analysis comparing the effects of different antibiotics on mortality in patients with BSIs caused by Enterobacterales-producing chromosomal AmpC β-lactamases, no strong evidence exists to suggest that piperacillin-tazobactam, cefepime or quinolones are inferior to carbapenems (meropenem or imipenem), provided that they are susceptible.^36^ Notably, there are no comparative data evaluating outcomes of ertapenem treatment for infections with AmpC-producing Enterobacterales.

Piperacillin-tazobactam (at a MIC ≤ 8 mg/L) and cefepime (at a MIC ≤ 2 mg/L) offer a carbapenem-sparing opportunity as directed therapy of susceptible Amp-C organisms, particularly for those Enterobacterales that have a low risk for clinically significant over-expression of AmpC (< 5%) such as *S. marcescens, M. morganii* and *Providencia* spp. In *E. cloacae* and *K. aerogenes* infections, de-escalation to piperacillin-tazobactam or cefepime may be considered once the MIC is known.

Therefore, it is reasonable to base treatment decisions for AmpC producers on:

- *In vitro* susceptibility
- Site of infection
- Clinical status
- Enhanced drug dosing and administration strategies
- Obtaining adequate source control
- Close monitoring to evaluate clinical responses.

Regarding the new BLICs, clinical data regarding the efficacy of CT and CA in the treatment of infections caused by AmpC- producing isolates are limited.^37^ Ceftolozane-tazobactam and CA have similar spectra of antibacterial activity, but with some important differences. The key microbiologic difference is that avibactam inhibits carbapenemases, particularly KPC and OXA-48, while tazobactam does not. Notably, although tazobactam does not inhibit Amp-C β-lactamases, recent data have demonstrated that CT is active *in vitro* against 99.7% and 94.7% of isolates with moderately and strongly up-regulated efflux mechanisms respectively, and also against 96.6% of bacteria with de-repressed AmpC, confirming earlier data that ceftolozane may overcome the two most prevalent mechanisms of resistance (up-regulated efflux and de-repressed AmpC) in *P. aeruginosa*.^38^ While both CT and CA have been shown to be microbiologically and clinically effective in ESBL, AmpC and *P. aeruginosa* infections,^39,40,41^ from a stewardship point of view, the specific efficacy of CT against MDR SPICE bacteria, upon failure of preferred agents, is important (Table 3).

In a global cohort of CR *P. aeruginosa*, including isolates from one centre in Cape Town, South Africa, CA was the most active agent with 72% susceptibility as per CLSI breakpoints compared with 63% for CT.^14^ In the cohort, 87% were genotypically positive for a carbapenemase with the most common being the MBLs; VIM most frequently followed by class A GES, which explains the relatively lower CT activity. In this regard, comparative CT antibiograms for CR-*P. aeruginosa* in South Africa should be performed, to ascertain geographic differences in non-susceptibility and inform potential use. In the study, 46% of isolates remained susceptible to both ceftazidime and cefepime.^14^ Notably, 90% and 86% of carbapenemase-negative CR isolates remained susceptible to CA and CT, respectively.^14^

Soon after registration in the United States of America, several studies reported the *in vivo* emergence of CT resistance in MDR-*P. aeruginosa*, associated with *de novo* mutations, rather than by acquisition of resistance from nosocomial isolates.^42,43^ Further studies are, therefore, necessary to optimise and validate administration techniques designed to minimise emergence of resistance, such as infusion strategies derived from PK/PD data, and to verify the mechanism by which resistance occurs *in vivo*.^42,44^

Overall, the data as presented seem to support the preferred stratified and strategic use of CT in the management of serious infections with AmpC producers in the absence of MBLs as directed therapy particularly for DTR-*P. aeruginosa* (Figure 1, Table 3).

## Carbapenemase-producing Enterobacterales

The clinical activity of CA against MDR-Enterobacterales and *P. aeruginosa* isolates, pooled from the various adult Phase III clinical trials (complicated intra-abdominal infection [cIAI] [RECLAIM 1 and 2; RECLAIM 3], complicated urinary tract infection [cUTI] [RECAPTURE 1 and 2], hospital-acquired pneumonia [HAP], including ventilator-associated pneumonia [VAP] [REPROVE] and cIAI or cUTI caused by ceftazidime-non-susceptible GNB [REPRISE]) demonstrated, that CA has similar clinical efficacy to predominantly carbapenem comparators against MDR Enterobacterales and *P. aeruginosa*.^45^

Ceftazidime-avibactam may improve outcomes among patients with CRE infections and appear to be superior to conventional therapies, including colistin, against BSI caused by KPC with clinical cure in the majority of DTR infections that had failed previous therapy.^46,47,48,49,50^ Qualitative evidence of successful use of CA for treatment of hospitalised patients with GNB infections with limited treatment options, based on clinical and microbiological outcomes and mortality, including evidence of effectiveness against CRE and MDR-*P. aeruginosa*, were recently reviewed.^51^

Ceftazidime-avibactam also has more favourable PK characteristics than colistin and has been shown to be better tolerated and several positive cost-effectiveness analyses have also been reported for BSIs, HAP and VAP.^52,53^ Outcomes evaluated included quality-adjusted life-years, healthcare costs and incremental cost-effectiveness ratios. These studies found that CA was cost-effective for CRE-BSIs and -pneumonia based on accepted willingness to-pay-standards in both the United States (US) and Italy.^52,53^ Similar conclusions were reached for low- to middle-income countries for CA versus colistin for CRE-BSIs and pneumonia in Peru.^54^

Particularly relevant to South Africa, recent reports have focused on the role of CA in OXA-48-producing bacteria.^55,56^ Avibactam is a diazabicyclooctane (DBO), which is a non β-lactam β-lactamase inhibitor that may have the ability to inhibit OXA-48 β-lactamases by forming a stable covalent complex. Although combination therapy is thought to improve the likelihood of clinical cure and survival in severe CPE infections, successful outcomes have been seen in approximately 70% of patients with infections caused by OXA-48-producing Enterobacterales treated with CA monotherapy.^55,56^ A recent review confirmed no significant differences in mortality in the treatment of CRE infections with CA-combination therapy compared to CA-monotherapy.^57^ Ceftazidime-avibactam thus shows promising results as monotherapy, and of the BLICs, is currently the preferred agent for the treatment of patients with severe infections because of OXA-48-producing Enterobacterales.^58^

Several studies have demonstrated *in vivo* development of resistance following relatively short courses (10–19 days) of therapy for KPC-BSIs.^59,60^ For CA, resistance mostly occurs in *K. pneumoniae* ST258 harbouring D179Y substitutions in the KPC enzyme, and proliferation of these KPC variants threatens its future use.^61,62,63^ Notably, reports on the rise of resistance to CA are alarming, not only in *K. pneumoniae*, but recently among other common Enterobacterales species. Of major concern, in the South African private sector CA non-susceptibility in CR-*K. pneumoniae* ranges between 10% and 20% and increases to 38% in those isolates causing BSIs where concurrent OXA-48, NDM, or VIM carbapenemases are present (Data on file).

In line with the proposed conceptual approach to BLIC therapy (Figure 1) and ‘Best practices’ for use of existing and new antibiotic options (Table 4), CA is recommended as directed therapy for severe OXA-48 producing infections, on bacteriological or clinical failure of preferred agents.

## Conclusion

The introduction of CT and CA into hospital formularies should be considered with great care, preferably with pre-planned stewardship-guided interventions emphasising their positioning as therapeutic options (Box 1). The focus on appropriate stewardship practices is vital to maximise the efficacy and longevity of all new agents that enter clinical practice. Preferably, no hospital should be allowed to implement the use of these drugs without the oversight of an infectious disease specialist, an experienced intensivist, microbiologist and pharmacists ABS teams. These new agents are generally not suitable for empiric use and as such is discouraged. Other clinical management challenges relate to uncertainty as to whether the new BLICs should be used as mono- or combination therapy.

BOX 1 Summary of recommendations for ceftazidime-avibactam and ceftolozane-tazobactam.

**Key antibiotic stewardship principles**

- Ceftazidime-avibactam and ceftolozane-tazobactam are indicated for DTR Gram-negative bacterial infections, defined as treatment limiting resistance that is, all β-lactams, including carbapenems and β-lactamase inhibitor combinations and fluoroquinolones, in the following indications:
  ■ Complicated IAI
  ■ Complicated UTI, incl. pyelonephritis
  ■ HAP, VAP
  ■ BSIs
- Ceftazidime-avibactam and ceftolozane-tazobactam are antibiotics of last resort and empirical use is discouraged. No hospital should be allowed to implement use of these drugs without oversight of either an ID specialist, microbiologist, an intensivist experienced in ABS, or pharmacist teams
- Recommendations for appropriate duration: Procalcitonin guided duration of therapy is advised where resources are available, otherwise, duration generally should not exceed the following^64^:
  ■ Primary GNB BSIs: 7 days
  ■ HAP: 5 days (*P. aeruginosa* 7 days)
  ■ Catheter-associated UTIs: 7 days
  ■ IAI: 4 days with adequate source control

**Recommendations for the appropriate prescribing of ceftazidime-avibactam**

In order to preserve the activity of ceftazidime-avibactam, it is the recommendation of the panel to restrict its use to the treatment of serious infections caused by DTR CRE that is, resistant to all β-lactams, including carbapenems and fluoroquinolones. This includes the following:

- Serious infections caused by OXA-48-like, KPC or GES CRE-GNB isolates where the MIC of the class 2 carbapenems (imipenem, meropenem) are ≥ 8 µg/mL, and ceftazidime-avibactam tests indicate susceptibility
- Serious infections caused by OXA-48-like- KPC or GES CRE-GNB where a patient has clinically failed combination carbapenem therapy, in spite of adequate source control
- Ceftazidime-avibactam in combination with aztreonam for treatment of serious infections caused by organisms that test positive for OXA-48-like enzymes as well as metallo-β-lactamases such as NDM, VIM and IMP?

**Recommendations for the appropriate prescribing of ceftolozane-tazobactam**

In order to preserve the efficacy of ceftolozane-tazobactam, it is the recommendation of this panel to restrict its use to the following infections where ceftolozane-tazobactam tests susceptible, in the absence of other options:

- Treatment of critically ill patients with HAP or VAP, who have confirmed DTR *Pseudomonas aeruginosa* or SPICE *Enterobacterales*
- Complicated IAI with DTR *P. aeruginosa* and/or SPICE *Enterobacterales*
- BSIs with DTR *P. aeruginosa* or SPICE *Enterobacterales*

DTR, difficult-to-treat resistant; IAI, Intra-abdominal infections; UTI, Urinary tract infections; HAP, Hospital-acquired pneumonia; VAP, Ventilator-associated pneumonia; BSIs, Bloodstream infections; ID, Infectious disease; ABS, Antibiotic stewardship; GNB, Gram negative bacilli; CRE, Carbapenem-resistant *Enterobacterales*; OXA-48, Oxacillinase-48; KPC, *Klebsiella pneumoniae* carbapenemase; GES, Guiana-Extended-Spectrum β-lactamase; NDM, New-Delhi metallo-β-lactamase; VIM, Verona Integron-encoded metallo-β-lactamase; IMP, Imipenemases; SPICE, Serratia spp., *P. aeruginosa*, Indole-positive Proteeae (*Morganella morganii, Providencia stuartii* and *Providencia rettgeri*), *Citrobacter* spp., *Enterobacter* spp.

† The management approach to the dual carbapenemase enzyme positive Enterobacterales is not entirely clear and a paucity of data exists in this regard.

The emergence of antibiotic resistance after short courses of therapy with CT and CA highlights the importance of establishing strict criteria for the use of these drugs and the continuing need for new antibiotics. Moreover, it substantiates the fact that these antibiotics at the current time, are the archetypal ‘antibiotics of last resort’. It further emphasises the ongoing challenges of treating infections caused by DTR-GNB species, and the substantial threat that resistance poses to other novel BLICs and future drug development. ‘An antibiotic steward knows how to use an antibiotic, a good antibiotic steward knows when to use an antibiotic, and a great antibiotic steward knows when not to use an antibiotic’ (Adapted from a quote regarding surgeons^65^).
